# Iron, Zinc and Phytic Acid Retention of Biofortified, Low Phytic Acid, and Conventional Bean Varieties When Preparing Common Household Recipes

**DOI:** 10.3390/nu12030658

**Published:** 2020-02-28

**Authors:** Marijke Hummel, Elise F. Talsma, Victor Taleon, Luis Londoño, Galina Brychkova, Sonia Gallego, Bodo Raatz, Charles Spillane

**Affiliations:** 1 Plant and AgriBiosciences Research Centre (PABC), Ryan Institute, National University of Ireland Galway, University Road, H91 REW4 Galway, Ireland; marijke.hummel@wur.nl (M.H.); galina.brychkova@nuigalway.ie (G.B.); 2 Division of Human Nutrition and Health, Wageningen University, 6708 PB Wageningen, The Netherlands; elise.talsma@wur.nl; 3 HarvestPlus. c/o International Food Policy Research Institute (IFPRI), Washington, DC 20005-3915, USA; v.taleon@cgiar.org; 4 International Center for Tropical Agriculture (CIAT), Cali 763537, Colombia; l.londono@cgiar.org (L.L.); s.gallego@cgiar.org (S.G.); b.raatz@cgiar.org (B.R.)

**Keywords:** micronutrients, plant, beans, nutrition, anti-nutritionals, biofortification, cooking, retention, Phaseolus vulgaris, *lpa*, SDG2

## Abstract

Biofortification is an effective method to improve the nutritional content of crops and nutritional intake. Breeding for higher micronutrient mineral content in beans is correlated with an increase in phytic acid, a main inhibitor of mineral absorption in humans. Low phytic acid (*lpa*) beans have a 90% lower phytic acid content compared to conventional beans. This is the first study to investigate mineral and total phytic acid retention after preparing common household recipes from conventional, biofortified and *lpa* beans. Mineral retention was determined for two conventional, three biofortified and two *lpa* bean genotypes. Treatments included soaking, boiling (boiled beans) and refrying (bean paste). The average true retention of iron after boiling was 77.2–91.3%; for zinc 41.2–84.0%; and for phytic acid 49.9–85.9%. Soaking led to a significant decrease in zinc and total phytic acid after boiling and refrying, whereas for iron no significant differences were found. *lpa* beans did not exhibit a consistent pattern of difference in iron and phytic acid retention compared to the other groups of beans. However, *lpa* beans had a significantly lower retention of zinc compared to conventional and biofortified varieties (*p* < 0.05). More research is needed to understand the underlying factors responsible for the differences in retention between the groups of beans, especially the low retention of zinc. Combining the *lpa* and biofortification traits could further improve the nutritional benefits of biofortified beans, by decreasing the phytic acid:iron and zinc ratio in beans.

## 1. Introduction

Iron and zinc deficiencies are amongst the most common micronutrient deficiencies globally and are estimated to affect over 2 billion people [1,2,3]. These deficiencies are associated with anemia (iron) [4] and impaired immunity and development (zinc) [5] and lead to major losses of human potential [6,7]. A significant part of the population that is suffering from micronutrient deficiencies consume beans as part of their daily diet, especially in Latin America and Eastern Africa [8]. Diets of rural and poor populations in these regions are mostly plant-based, in which legumes (and more specifically beans) are an essential component of daily diets [9]. Common beans (*Phaseolus vulgaris* L.) are an excellent source of not only iron and zinc but also proteins, dietary fiber, and vitamins [10].

Biofortification, a nutrition-sensitive agricultural intervention, aims to improve the nutritional status of resource-poor populations through increasing the nutrient content of food crops, by developing more nutrient-rich crop varieties [11]. HarvestPlus, a global interdisciplinary alliance of research and implementing agencies engaged in biofortification, use conventional breeding to improve the nutritional quality of staple crops without compromising other agronomic qualities (e.g. yield, drought resistance, etc.) [12]. Iron beans are biofortified lines of beans with increased levels of iron and zinc that have been developed by HarvestPlus and have been released in 18 countries in Latin America and 26 countries in Africa [13]. Micronutrient targets for breeding biofortified crops are established based on the food intake of target populations, nutrient losses during storage and processing, and bioavailability of the target nutrient to the human body [14]. Current breeding targets for iron beans are 94 μg·g^−1^ compared to an average of 50 μg·g^−1^ as the baseline content of conventional varieties of beans [12].

Studies conducted to date on the iron bioaccessibility and bioavailability from (iron biofortified) beans have been using Caco-2 cell models, in vitro digestion models [15,16,17,18,19,20,21], poultry studies [16,20,21,22,23,24], and human feeding trials [25,26,27,28]. These studies show the influence of specific polyphenols on iron bio-accessibility and bioavailability depending on the type of bean. Furthermore, the positive effects of biofortified beans on iron status and other nutritional and functional indicators in humans are described. Mineral absorption from plant foods is generally low, which is mainly due to limited bioavailability of the iron and zinc to the body [29]. In particular, anti-nutritional compounds hamper the potential nutritional impact of consuming plant foods and iron beans, specifically [30]. Examples of such anti-nutritional compounds are phytic acid, polyphenols, lectins, and tannins.

Current research suggests that phytic acid is one of the major and significant inhibitors of mineral bioavailability from beans, next to polyphenols [8]. Phytic acid (myo-inositol-1,2,3,4,5,6-hexakisphosphate) and its salt phytate are known for their negative effect on iron absorption and can decrease iron status [8]. Phytic acid is the main storage form of phosphorus and mineral storage in the bean seed and plant. It has been demonstrated that reductions in phytic acid levels in beans are not associated with reduced plant health or yields [30,31]. Hence, it is possible to develop low phytic acid (*lpa*) beans, with preferable agronomic traits.

For micronutrient biofortification strategies to successfully impact on human nutrition, sufficient levels of retention of target micronutrients after typical processing, storage, and cooking practices must be demonstrated [32]. Also, mineral absorption of the biofortified crops should be similar or better than non-biofortified crops. However, absorption of iron and zinc in biofortified crops could be limited by its antinutrient content, such as phytic acid. In the case of beans, common processing techniques include soaking, boiling, and refrying. Micronutrients are lost in preparation methods due to chemical degradation (isomerization and oxidation) and physical loss, through the leaking of soluble solids into water or water loss [32]. For instance, soaking has been shown to reduce phytic acid by solubilizing them in the soaking water, while on the other hand, it can also cause leaching of minerals [33]. Micronutrient losses during food processing and cooking can be measured by determining True Retention (TR), where the changes in solids of food during processing and cooking are taken into account, to provide an accurate estimation of actual retention during the different processes [32]. Retention studies that have tested conventional [34,35,36] and biofortified beans [32] have been published. However, the studies to date have not reported TR, which makes it difficult comparing results across different studies.

Low phytic acid mutant lines have been developed using a mutant allele of a gene that prevents the storage of phytic acid in the bean [30]. Whereas research has been conducted to study retention in conventional bean varieties, no research on retention levels in these relatively new *lpa* lines has been published. If these more freely available or weakly bound minerals are retained in beans while being processed, this could provide a route for further development of biofortified beans that combine high mineral and *lpa* traits. Therefore, we aimed to assess the iron, zinc and total phytic acid levels of *lpa*, biofortified and conventional beans and evaluated the iron, zinc and phytic acid retention when preparing common bean recipes using the different classes of bean varieties.

## 2. Materials and Methods

### 2.1. Bean Groups and Varieties

Seven different varieties of three different groups of common beans (biofortified, *lpa* and conventional) were selected for this study. These included three biofortified varieties (BIO101, BIO107 and ICTA Chortí), two genotypes carrying the *lpa* mutation, and two conventional bean varieties (Caraota and breeding line DAN20 of the Calima grain type). These were two black bean grain types, two *lpa* lines with medium/small brown grain, two medium/small red grain types, and one Calima type variety. The control varieties were commonly used bean types grown and consumed in South America and Eastern Africa. The low phytic acid lines *lpa-1* and *lpa-2* were generated at the International Centre of Tropical Agriculture (CIAT), Colombia, from the bean line *lpa*-127-4, which is a BC2 (backcross 2) line arising from the backcrossing of the original *lpa* mutant line *lpa 280-10* (a homozygous monogenic recessive *lpa* mutant line obtained by EMS mutagenesis, [30]) with the bean cultivar BAT93. The line *lpa*-127-4 was further backcrossed to BAT93 and the two *lpa* lines, *lpa-1* and *lpa-2*, were selected as having the lowest phytic acid content from the plant lines screened across the BC2F4 generation. The pedigree that led to the *lpa-1* and *lpa-2* bean lines used in this study is detailed in supplementary Figure S1. BIO 101 and BIO 107 are biofortified varieties that were released in 2016 in Colombia [37,38]; ICTA Chortí was released in Guatemala in April 2017 [39].

All varieties were grown in Valle del Cauca, Colombia and harvested between October 2016 and March 2017. Exceptions were the black bean variety ICTA Chortí, which was imported from Jalapa, Guatemala, and Caraota, which was bought from a supermarket in Cali, Colombia. In Table 1, a description of all genotypes and their characteristics is provided. Beans were dried and stored in a cold room (10 °C) until further processing.

### 2.2. Cleaning Procedure for Beans and Materials

Dry beans were cleaned by removing any dirt, disease-infected beans and any beans with a broken seed coat. After weighing, the seeds were cleaned using ultrapure water (18 MΩ) (MilliQ^®^ Merck-Millipore, Darmstadt, Germany), drained and dried using paper towels for sampling, or used straight away for preparing the recipes. All materials used for sample preparation were decontaminated from free minerals by overnight bathing in a 5% HCl solution with ultrapure water (18 MΩ). All recipes and bean cultivar combinations were prepared in duplicate and sampled at every stage, as described below. All processing and cooking of bean samples was performed at CIAT in Cali, Colombia.

### 2.3. Cooking and Sampling Procedure

**Figure 1:**
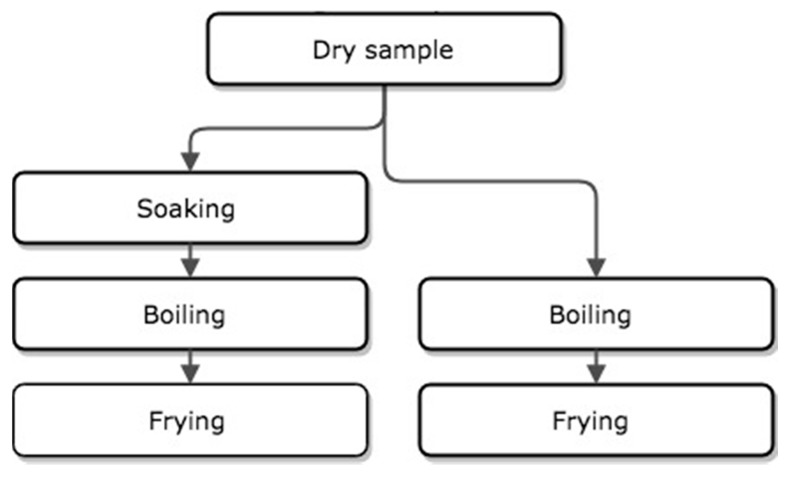
Overview of the study design with the different bean preparation methods.

Two different recipes of beans were prepared; boiled and refried beans, using either soaked or dry beans (Figure 1). Samples for analysis were taken during all of the steps described below, cooled down, and stored in an −80 °C freezer until further processing. Samples for ICP-MS and phytic acid analysis were freeze-dried (Labconco, FreeZone, Kansas, MO, USA). Scales used were Scout Pro, models PRO SP6000 and PRO SP402 (Ohaus, Parsippany, NJ, USA). Weights were recorded at each step, both before preparation and the finalized product.

#### 2.3.1. Soaking Procedure

Three-hundred grams of dry beans were added to 1500 mL of MilliQ (1:5) water in a glass beaker and soaked at room temperature for 18 h. Beans were drained, and samples of soaked beans and the soaking water were taken. The equivalent of 200 g of dry beans was taken to the next step for boiling.

#### 2.3.2. Boiling Procedure

For boiling, 200 g of dry beans or the equivalent of soaked beans was added to 1500 mL Milli-Q (1:7.5) in a glass beaker for cooking on a pre-heated electrical plate (350 °C, Corning, model PC-620D, New York, NY, USA). Total cooking time ranged from 37 to 90 min, depending on the variety. Beans were cooked until they felt soft between fingers, after which they were drained. Samples were taken after cooling down the broth and the beans for 30 min at room temperature. The equivalent of 100 g of dry beans was taken to the next step for refrying.

#### 2.3.3. Refrying Procedure

A standardized recipe of refried beans was prepared using boiled beans. The equivalent of 100 g of dry beans, as boiled beans, was mixed with 200 g of cooking broth and 20 g of canola oil (brand Premier, Lloreda) and blended (using Osterizer model 4655, stainless steel, Oster, Mexicali, Mexico) for two repeated periods of 1 min., after which the mass was added. The resulting mass was placed in a Teflon pan, which was then preheated for 1 min on a hot stove to an average of 210 °C and continuously stirred until enough water was evaporated to form a firm mass covering around half of the pan. The mass was turned until both sides were cooked, and a light brown crust appeared. This took on average 10 min and 30 sec. Samples were taken immediately after.

### 2.4. Cooking Time Determination

Cooking times were determined using an automated Mattson cooker (Mattson, Winnipeg, Canada), as described by Wang et al. [40]. The cooker consists of 25 stainless steel piercing rods that are placed on top of 25 soaked (16 h at room temperature) bean grains. The whole device is placed into a 2 L glass beaker containing 1 L of boiling MilliQ^®^ water heated using an electrical heating plate (Waring Pro Extra Burner, SB30, Amarillo, TX, USA). The grain is considered cooked when the rod penetrates and touches a metal disc under it; at this moment, the time is automatically recorded for each of the grains. Cooking time is defined as the number of minutes required for 80% of the samples to be pierced.

### 2.5. Iron and Zinc Analysis

Iron and zinc were measured by Inductively coupled plasma mass spectrometry (ICP-MS) (7500cx; Agilent Technologies, Santa Clara, CA, USA), at Flinders University, Australia. All seed samples were gamma-irradiated at 50 kGray for sterilisation prior to release into Australia. Prior to grinding, samples were dried thoroughly at 80 °C for at least 12 h, after which samples were placed in a desiccator to keep the samples dry. Samples were ground to a flour using a Retsch Ultra Centrifugal Mill ZM 200 fitted with a 12-tooth titanium rotor, titanium sieve, and pan (Retsch GmbH & Co KG, Haan, Germany). Ground samples were again dried at 80 °C for at least 12 h and put in a desiccator until further analysis. A closed-tube digestion method was used for digesting samples [41]. All samples used for the validation and calibration contained <4 mg/kg Al, indicating these samples can be considered free from soil contamination as per HarvestPlus guidelines [42].

### 2.6. Phytic Acid Analysis

Phytic acid (IP_6_) and lower myoinositol phosphates (IP_-1/2/3/4/5_) content was measured based on a modified procedure of Latta and Eskin (1980) using polyprep prefilled chromatographic columns (Bio-Rad Laboratories, Richmond, CA, USA) containing an AG-1-X8 anion exchange resin (100–200 mesh chloride form, 0.8 × 4 cm), allowing isolation of phytic acid from bean extract. Briefly, the bean sample (0.5 g, 1.0 g for *lpa* samples) was extracted with 0.65M HCl (20 mL) for 2 h. After centrifugation (3800 RPM, 15 min), 2 mL of the supernatant was added to the column (8 mL for *lpa* samples). Interfering compounds and inorganic phosphorus were removed by washing with ultrapure water (18 MΩ, 5 mL) followed by 0.07 M NaCl (10 mL). Bound phytic acid (IP_6_) and IP_-1/2/3/4/5_ was eluted with 0.7 M NaCl (30 mL), and an aliquot of the eluate (0.9 mL) was vortexed with 0.3 mL of Wade reagent (0.03% iron(III) chloride, 0.3% sulfosalicylic acid). Absorbance of the salicylate–Fe(III) complex was measured at 500 nm using a spectrophotometer (BioTek Instruments, Inc. Winooski, Vermont, USA). The concentration of phytic acid was calculated from a prepared standard curve obtained with potassium phytate and it was assumed that all phosphorus measured was released from IP_6_ (0–60 mg/mL; Sigma-Aldrich Canada, Oakville, ON, Canada) [43].

### 2.7. Statistical Analyses

True retention (TR) for all samples at all processing steps was calculated. TR takes into account loss of dry mass (i.e., soluble solid losses and dry matter losses due to preparation) over the process. TR was calculated using Equation (1), where Nc = nutrient content per g of cooked food, Wc = weight of cooked food (g), Nr = nutrient content per g of raw food, and Wr = Weight of food before cooking (g). Meanwhile, apparent retention (AR) was calculated for the final products. Apparent retention (AR) does not take into account losses of dry matter during processing, and for this reason, it could be calculated if dry matter of food before and after cooking are unavailable. AR was calculated on a moisture-free basis, using Equation (2). TR is a more accurate method for calculating micronutrient retention compared to AR [32,44].

TR (%) = (Nc × Wc) / (Nr × Wr) × 100(1)

AR (%) = [Nc (dry weight basis)] / [Nr (dry weight basis)] × 100(2)

All statistical analyses were conducted using IBM SPSS statistics for Macintosh, version 23.0.0.2 (IBM Corp., Armonk, NY, USA) and RStudio version 3.6.1 (RStudio Inc., Boston, MA, USA). To perform ANOVA analysis, first we validated all six assumptions that are required for a one-way ANOVA to give a valid result. To validate that variables are normally distributed for every groups, we used “ggdensity” function in “ggpubr” R package to visualize the distribution of the data and performed skewness test for all variables to identify if transformation is required. If required, samples were transformed based on maximum-likelihood estimation of the power lambda and validated using “bestNormalize” package based on the best Pearson P / df values (close to 1), followed by subsequent skewness test, and normality distribution plot with Q-Q plot for standardized residuals for each variable as a function to variety or group (“MASS” package in R). The outliers were tested in “car” package, and if required, were removed based on the interquartile range method. The homogeneity of variance was analyzed using Bartlett’s test and Levene’s test, and validated by Welch test (“onewaytests” package) with a significance level of 0.05. The dependent variances (e.g. iron content, zinc content, phytic acid content, etc) were considered in respect to genotypes and plant groups. Normalized data was processed using analysis of variance (ANOVA) for variety, group and/or processing type followed by quantiles for residuals test using “lme” package in R. Post hoc analyses were done using Tukey’s test in “multicomp” package, and *p*-values < 0.05 were considered statistically significant. To validate the results of AVOVA analysis, we performed General Linear (GL) Analysis and Linear Mixed Model (LMM) analysis in R. The General linear (GL) analysis followed by multiple comparison of means by Tukey contract (Posthoc) was performed in the “nlme” package via “lme” function after contrasting with fixed variables and analyzing residuals. The p-value were adjusted to a single-step method. The Linear Mixed Model (LMM) analysis was performed in “emmeans package”. The contrasting pairs were analysed using pairwise multiple comparison of means by Tukey contract (Posthoc) at a confidence level of 0.95.

## 3. Results

### 3.1. Mineral and Phytic Acid Content of Dry Beans

The iron, zinc and phytic acid content of the dried bean grains are presented in Table 2. The average iron content for the biofortified varieties was 88.5 μg·g^−1^, well above the average of the other two groups of conventional and *lpa* beans (57.4–74.5 μg·g^−1^), but below the current breeding targets of 94 μg·g^−1^ for high iron beans [12]. The Calima variety contained the lowest levels of iron with 54.4 μg·g^−1^, whereas the BIO101 variety contained the most iron (90.2 μg·g^−1^). Within the groups of biofortified and *lpa* beans, the bean varieties did not significantly differ in iron levels. For the conventional varieties, there was a significant difference between the two varieties (*p* < 0.05).

For zinc content, the average of the biofortified varieties was 39.1 μg·g^−1^ compared to 30.2–31.1 μg·g^−1^ for the non-biofortified and *lpa* varieties. The *lpa*-1 variety contained the least zinc (28.7 μg·g^−1^), whereas the BIO101 variety contained the highest level of zinc (43.5 μg·g^−1^). The correlation between iron and zinc levels in these beans is *r* = 0.76 (*p* < 0.05).

Total phytic acid levels in the biofortified varieties were on average 18.6 mg·g^−1^ compared to 16.1 mg·g^−1^ in the conventional varieties (no significant difference). Research indicates that an increased iron content is correlated with an increased phytic acid content [45]. The *lpa* varieties contained on average 1.1 mg·g^−1^ of phytic acid, which is only ~6% of phytic acid compared to the conventional varieties, these are comparable levels to those previously reported in other studies on *lpa* beans [30,31,46].

### 3.2. Cooking Times

Cooking times ranged from 33.8 min for the BIO101 variety to 62.7 min for the ICTA Chorti variety as shown in Table 3. The average cooking time was 54.0 min. Both biofortified varieties had the shortest cooking time. The smallest bean genotypes (*lpa* lines and Chorti) had a larger standard deviation compared to the other bean varieties. Cooking times were only determined in soaked grain because with this cooking time determination method, the non-soaked seeds were slipping away under the piercers of the Mattson cooker.

### 3.3. Nutrient Retention in Soaked, Boiled and Refried Beans

#### 3.3.1. Iron

Table 4 presents an overview of the iron retention in different groups of beans. After soaking, TR values ranged from 98.8 to 108.4%. TR in conventional varieties was significantly higher compared to the *lpa* and biofortified varieties (*p* < 0.05). Iron levels after soaking ranged from 27.5 to 39.1 μg·g^−1^ for fresh weight (FW) and 64.2 to 91.2 μg·g^−1^ based on dry weight (DW) (Table A1). TR values after boiling beans were 77.2–91.3%, whereas AR values were 104.8–119.6%. Conventional varieties had a significantly higher AR and TR compared to the *lpa* varieties. Biofortified varieties had a higher AR and TR compared to the *lpa* varieties after boiling, but this was not always significant (*p* > 0.05). Iron levels after boiling were 21.4–33.0 μg·g^−1^ in FW and 59.6–90.8 μg·g^−1^ DW (Table A1). TR values after refrying beans were 87.3–104.5%, whereas AR values were 91.4–100.5%. Conventional beans had a significantly higher TR than the biofortified and *lpa* beans after refrying of non-soaked beans (*p* < 0.05). Iron levels after refrying were ranging from 18.0 to 27.8 μg·g^−1^ in FW and 48.9–74.5 μg·g^−1^ in DM. There were no significant differences found in the TR or AR for iron between soaked and non-soaked beans for both boiled and refried (*p* > 0.05). Thus, soaking does not influence iron levels when boiling or refrying beans.

Overall, the iron loss after processing was low. In this study, we found an average loss of 16% after boiling and a 9% loss after refrying the beans based on TR. No major differences were found between the different groups of beans, indicating that the *lpa* beans do not show a different pattern in retention compared to biofortified or conventional beans concerning iron retention.

The generalized linear mixed model highlighted an interaction between the group of beans and type of treatment as the main explanatory factors for true retention and apparent retention of iron.

#### 3.3.2. Zinc

Table 5 presents the zinc retention in different groups of beans. After soaking, TR values ranged from 93.3 to 99.4%. On the group level, no significant differences in TR were found after soaking beans. Zinc levels after soaking were 11.7–16.5 μg·g^−1^ in FW and 30.1–38.5 μg·g^−1^ in DM (Table A2).

TR values after boiling beans ranged from 41.2% to 84.0%, whereas AR values were between 49.3% and 96.2%. After boiling, a decrease of ~50% was found in the TR and AR of *lpa* varieties. No differences were found in the TR and AR between conventional and biofortified varieties. Zinc levels after boiling ranged from 5.4 to 14.0 μg·g^−1^ in FW and 14.9–37.5 μg·g^−1^ in DM. TR values after refrying beans were 63.5–100.0%, whereas AR values were 58.1–81.4%. Both groups of soaked and non-soaked *lpa* beans had different (*p* < 0.05) AR and TR compared to the conventional and biofortified varieties. The highest difference was in TR after refrying and soaking; a 23% lower retention was recorded in *lpa* beans compared to conventional beans. Zinc levels after refrying were 6.4–12.3 μg·g^−1^ in FW and 17.6–31.5 μg·g^−1^ in DM.

Refrying increased the TR for zinc to an average of 85% for the soaked beans and 95% for the non-soaked beans (*p* < 0.05). A significant difference was found in TR after soaking the beans for both boiling and refrying (*p* > 0.05), where we found a higher retention in the non-soaked beans (data not shown).

Overall, we can conclude that zinc retention is low when compared to iron retention. An average of 4.6% of zinc is lost during soaking. However, during boiling, retention is very low for the *lpa* varieties (average loss of 56%), especially in comparison with the conventional and biofortified varieties (average loss of 20%). After refrying, zinc retention is increased but still lower in the *lpa* beans, (29% loss) compared to the other varieties (9% loss) (*p* < 0.05). Indeed, the generalized linear mixed model highlighted the importance of a group effect on the overall model of zinc retention.

#### 3.3.3. Total Phytic Acid

Table 6 presents an overview of total phytic acid retention in different groups of beans. After soaking, TR values ranged from 65.6% to 88.5%. TR for phytic acid was significant lower for conventional beans compared to *lpa* and biofortified beans (*p* < 0.005).

Phytic acid levels after soaking were 0.4–7.0 mg·g^−1^ for FW and 1.0–16.0 mg·g^−1^ in DM. The TR values after boiling beans were 66.9–79.5%, whereas AR values were 75.5–93.4%. *lpa* beans have a significantly higher retention of phytic acid compared to the other varieties. However, the absolute levels of phytic acid are still about 10% of that in the other groups of beans. Soaking beans led to a significantly lower retention of phytic acid after boiling compared to non-soaked beans (*p* < 0.05) (data not shown). Phytic acid levels after boiling were 0.33–4.70 mg·g^−1^ for FW and 0.92–12.81 mg·g^−1^ in DM (Table A3).

The TR values after refrying beans were 59.7–86.9%, whereas AR values were 53.6–77.0%, which means a substantial loss of 13–40% of phytic acid. Refrying increased the zinc TR with an average of 7% compared to boiling. Phytic acid levels after refrying were 0.28–3.98 mg·g^−1^ for FW and 0.72–11.24 mg·g^−1^ in DM. An effect on both AR and TR through soaking was observed (*p* < 0.05), where the retention of phytic acid was lower after both boiling and refrying when the beans were soaked.

Overall, while we found a higher retention of phytic acid in the *lpa* beans compared to the other groups of beans, the *lpa* beans had very low phytic acid levels compared to the conventional and biofortified varieties. The lowest retention of phytic acid was found in the conventional varieties. Soaking helped to remove phytic acid, as demonstrated by a significantly lower TR phytic acid content when comparing soaked with non-soaked beans. Similarly to zinc, groups of beans had a major contribution to generalized linear model fit.

### 3.4. Contribution of Beans to the Estimated Average Requirement (EAR) of Iron and Zinc Intake

The contribution of beans to the mineral intake in populations with a regular bean consumption, either boiled or refried beans, was estimated for the different groups of beans. The *lpa* varieties of beans had a significantly higher zinc loss compared to the biofortified and conventional varieties. The differences in levels of iron and zinc, and TR, have an impact on the iron or zinc contribution to the Estimated Average Requirement (EAR) after consuming beans and depend on the preparation method used. The percentage contribution to the EAR was calculated considering an EAR of 4.1 mg d^−1^ of iron [47] and 4 mg d^−1^ of zinc for children aged 4–6 years old [48]. For adult women this was 8.1 mg d^−1^ for iron [47] and 7 mg d^−1^ for zinc [48]. The average FW iron/zinc content of soaked and non-soaked beans for each group of varieties was used. The average intake of dry beans in Rwanda was 107 g for children and 198 g for adults, which is among the highest in the world [49]. For easy comparison throughout different preparation methods, we assume and compare here an intake of 50 g of dry beans (~half cup, one portion), equivalent to 100 g of cooked beans, and 125 g of refried beans (based on our data). For children between 4 and 8 years old, we assume the portions are 55% compared to the adults, based on the Rwanda data. The contributions of the different groups of beans to the EAR of iron and zinc for children 4–8 years old and adult women can be found in Table 7. Results show that one portion could contribute up to 46% and 43% to the iron EAR for respectively children and adult women. For the zinc EAR, this is 21% for both children and adult women. In both cases, refried beans contribute slightly more to the EAR per portion, and biofortified beans are the best source among the three groups of beans.

### 3.5. Mineral-Phytic Acid Ratios of Beans under Study

The bioavailable fraction of iron and zinc from beans after consumption is the fraction that is contributing to the physiological function and/or storage in the human body [29]. Bioavailability of iron and zinc has shown to be negatively influenced by the amount of phytic acid in the meal and in the whole diet [50]. The phytic acid to mineral concentration relationship can be determined by calculating the molar ratios using the molecular weights of iron or zinc and phytic acid (MW = 660 g/mol). The phytic acid to mineral molar ratios for the beans under study are presented in Table 8 and demonstrate the very low ratios of 1:1 for the lpa beans compared to the conventional and biofortified varieties.

## 4. Discussion

Biofortification strategies to improve human nutrition require not only the development of biofortified varieties with high levels of micronutrients, but also of varieties that have lower levels of anti-nutritional compounds. Such anti-nutritional compounds can limit the bioavailability and uptake of micronutrients. In addition, for biofortified foods such as beans which are processed and cooked prior to consumption, it is essential that micronutrients are retained during the preparation of such foods in sufficient quantities to impact on human nutrition.

Here we demonstrate that the levels of iron and zinc found in the dry beans are comparable with those found in other studies [34,35,51]. We also detect a positive trend between iron and zinc levels, which has been observed by others [45,51,52]. Phytic acid levels found in conventional and biofortified beans in our study are also comparable to other studies, where phytic acid concentrations ranging from 4 to 26 mg·g^−1^ of beans have been reported [8,31,53,54].

We found that the cooking times assessed using the Mattson cooker showed a large variation in the cooking times of the *lpa* genotypes. Overall, the cooking time results should be interpreted with caution since storage time and temperature have been shown to influence cooking time. However, the cooking time was within the usual reported cooking times for beans [55,56].

Our iron retention results are comparable to a study with non-soaked beans in Rwanda that showed a retention close to 100% after boiling the beans. In the Rwandan study, cooking broth was not discarded, which prevented iron loss through the broth [32]. In contrast, in our study, the cooking broth was discarded, which led to a higher loss of iron. Carvalho et al (2012) found that iron retention for both soaked and non-soaked bean grains of six different common bean cultivars led to a loss of 13–19% of iron in non-soaked and soaked beans, which is similar to an average of 16% loss for both non-soaked and soaked beans in our study [34]. Refrying increased the iron TR, most certainly due to adding cooking broth to prepare the refried beans. This broth contained the iron that leaked into the cooking broth during boiling. To our knowledge, no other studies have reported iron retention after refrying beans. Values > 100% for AR as reported in our study were also reported before by Ongol et al. [36] and Ferreira et al. [35]. The high AR of > 100% for iron retention can possibly be explained by the leakage of solubles in the water (10.1–20.5%).

Retention of zinc was studied by Carvalho et al. and showed that zinc levels in broth after boiling beans did not differ between soaked or non-soaked beans [34]. Although we did not measure broth zinc concentrations, we did find a significant difference in zinc retention between soaked and non-soaked boiled beans; however, this difference was small. In addition, Carvalho et al. concluded that most zinc remained in the bean after boiling and was concentrated in the cooked bean [34].

Refrying increased the zinc TR up to 100% for conventional non-soaked beans, this was most likely due to adding the cooking broth to prepare the refried beans. This broth contained the zinc that leaked into the cooking broth during boiling.

The *lpa* bean genotypes showed substantial losses of zinc into the boiling water, which is partly reconstituted during refrying, where differences in retention are much smaller between the *lpa* and conventional group. No other studies have reported zinc retention after refrying beans. The higher affinity of zinc to phytic acid [57], the relatively high zinc amount trapped in the pericarp rich in phytic acid after soaking and steaming rice [58], and lower zinc retention in *lpa* beans during boiling soaked beans suggest that during soaking and cooking, zinc from the cotyledon in non-*lpa* beans possibly interacted with the phytic acid, preventing excessive zinc losses in the soaking and cooking water. However, phytic acid in *lpa* beans was found in relatively low quantities, and the zinc from these beans may not have interacted much with the limited amounts of phytic acid remaining, causing larger zinc losses in the soaking and cooking water. This possibility should be investigated further, not only for zinc, but also for iron because most iron is also found in the cotyledon of the bean [59] despite *lpa* beans having a different retention pattern compared to zinc.

Phytic acid levels were significantly reduced (> 10%) by soaking in our study. Another study in different types of Canadian pulses showed only a slight increase in phytic acid after soaking a black bean variety (2.34%) and pinto bean (1.86%). A decrease in phytic acid was found for a dark red kidney bean and a navy bean variety (−0.54% and −1.03%, respectively) [53]. A review of Haileslassie et al. compared 15 studies in which beans were soaked under various conditions. Results were ranging from no significant difference on phytic acid levels after soaking up to a 66% reduction in phytic acid after soaking in an autoclave [60].

In a study of Shi et al, cooking various bean varieties resulted in very modest decreases in phytic acid. Compared to the raw values for different types of beans, the decreases were between −2.29% and −0.29% [53]. This is very minimal in comparison with our study where phytic acid was reduced up to 50% after boiling. For the soaked samples, the soaking water was discarded and therefore higher losses of phytic acid were reported in comparison with the non-soaked beans when preparing boiled and refried beans. No other studies have published phytic acid retention after refrying beans.

Our analysis quantified the total amount of phytic acid in the samples, including other dephosphorylated forms of myoinositol with less phosphate groups (IP-_3/4/5_). These other compounds (especially the lower phosphorylated forms) do not necessarily inhibit mineral absorption to the same extent and therefore could lead to an overestimation of their actual effect. In vitro studies using Caco-2 cell lines demonstrated the inhibiting effect of phytic acid for different degrees of phosphorylation (IP-_3/4/5/6_) for both Fe and Zn [61]. IP_1-4_ were reported to not have an effect on zinc absorption in an animal study [62]. In a series of five human studies using extrinsic labelling, it was found that only inositol phosphates lower than IP_3_ had no effect on iron bioavailability [63]. Future research could further identify the type of phytic acid present in the different types of beans, as this might be another angle of explaining the differences in retention and eventually the effect on the bioavailability of minerals to the human body [50].

The molar ratios of phytic acid to iron found in this study are comparable to other studies where *lpa* beans were consumed by different groups of women to compare the iron bioavailability from different types of bean seeds [31,46]. Studies have shown that *lpa* beans have a higher iron bioavailability caused by the low concentration of phytic acid compared to conventional beans [31,46]. No data was found on the zinc bioavailability from *lpa* beans.

A multiple meal isotope bean study showed that both biofortified and *lpa* beans provided more bioavailable iron in comparison with conventional beans, however, there was no difference in fractional iron absorption [46]. In another single meal study, a 50–60% higher fractional absorption was found for *lpa* beans compared to conventional beans. In addition, it was reported that studies based on single meals often exaggerate the inhibiting effect of phytic acid on absorption of both iron and zinc [50]. One study used dephytinized beans (95% phytic acid reduction) and compared these to conventional and biofortified varieties for the fractional iron absorption in a multiple meal study [64]. Results showed a fractional absorption of iron of 13.2, 9.2 and 7.1% for respectively dephytinized, biofortified and conventional beans. When these results are extrapolated to the findings from our study, one portion of boiled beans could contribute for 14, 16 and 23% for respectively conventional, biofortified and *lpa* beans (taken as 95% dephytinized beans) of the physiological requirements of iron in an adult woman. Hence, the indications are that the *lpa* trait is promising and of public health relevance, especially in settings with a high iron deficiency prevalence, a high phytic acid diet, and a high consumption of beans.

In addition, the phytic acid content of the whole diet has shown to be of influence, particularly on the zinc bioavailability from beans. Absorption from diets with a phytic acid to zinc ratio of 12–15 compared to a ratio of 5 was approximately 50% less [65]. For iron, an increase in bioavailability influenced by phytic acid ratios is only found at very low ratios of 0.4–1.0 [66]. Hence, when *lpa* beans are used to replace conventional beans and would be added to an already low-phytic acid diet this could potentially increase the absorption of both iron and zinc significantly. Further research is needed to test to what extent low phytic acid-mineral ratios in beans can lead to a higher bioavailability of iron and zinc, when part of a whole diet.

The use of extrinsic labelling in determining the iron absorption and bioavailability has shown to not always be consistent when compared with intrinsic labelled foods, therefore, interpretation of these studies should be taken with caution [67]. Future studies should, where possible, be carried out with the use of intrinsically labelled foods to prevent these unwanted effects, or set up using in vitro digestion/Caco-2 cell models coupled with a poultry model that has also shown to be in strong agreement with human studies and a reliable tool for screening varieties [16,21].

The cotyledons contain 75–80% of iron, this location could potentially be the cause of the discrepancy between intrinsic and extrinsic labelling. The cotyledon cell walls represent a barrier for iron absorption from the bean, however, breaking these cell walls did not show an increase in the bioavailable fraction of iron. This suggests that the intracellular matrix of the bean potentially inhibits the exchange of iron with the cell transport mechanism [68].

The present study focused on total phytic acid content of beans and its possible effect on the bioavailability of iron and zinc. However, we recognize that polyphenols are an additional class of anti-nutritionals that need to be considered in high-Fe bean biofortification efforts and also with reference to the *lpa* trait. It has been shown in a series of in vitro digestion/Caco-2 cell models and/or coupled with poultry model studies that specific polyphenols in especially black beans inhibit iron uptake and that breeding for more iron in black beans does not lead to more bioavailable iron due to higher levels of polyphenolic compounds [19,20,24]. The overall inhibitory effect of polyphenols is combinatorial, whereby some polyphenols (catechin, 3,4-dihydroxybenzoic acid, kaempferol, and kaempferol 3-glucoside) promote iron uptake while others (myricetin, myricetin 3-glucoside, quercetin, and quercetin 3-glucoside) inhibit iron uptake.

As the *lpa* trait could be combined with different types and colors of beans, an optimal combination could be sought that has not only high mineral availability, but also good acceptability by consumers. One possible combination could be the yellow Manteca bean, which has shown to be fast-cooking and has a high iron bioavailability [17,22].

## 5. Conclusions

This is the first retention study on beans including *lpa* lines and comparing these with biofortified and conventional beans. Our results show a relatively high retention for the conventional and biofortified varieties after processing, consistent with literature. In contrast, *lpa* varieties have extremely low total phytic acid levels and a much lower retention of zinc, compared to the other groups of beans. More research is needed into the 1) binding of iron and zinc in the beans by phytic acid and 2) the types of phytic acid in the different groups of beans, 3) the retention of polyphenols, and the effect of these on the bioavailability of iron and zinc from the different types of beans. This will likely further explain our findings. Furthermore, our findings imply that soaking should be more widely promoted as a means to decrease total phytic acid content of beans, as this is likely to improve the bioavailability of iron and zinc.

There is no consensus yet on to what extent phytic acid and polyphenols influence the bioavailability of the minerals in the different types of beans and as part of a whole diet. However, different studies showed that lower phytic acid:iron/zinc molar ratios in beans have a higher fractional absorption of iron and, therefore, the *lpa* lines are promising in contributing to the iron and zinc intake.

Developing beans with an increased mineral content combined with a low phytic acid trait, low concentrations of specific polyphenolic compounds, and shorter cooking times could be the research target for the next generation of biofortified beans attractive for consumers and lead to a higher nutritional intake compared to the beans (including biofortified varieties) currently in the markets.

## Figures and Tables

**Table 1:** Description of included genotypes of beans.

Market Class (Seed Size)	Genotypes	Group
Calima—large red mottled	DAN20	Conventional
Red (medium/small)	BIO 101	Biofortified
Red (medium/small)	BIO 107	Biofortified
Black (small)	ICTA Chortí	Biofortified
Black (small)	Caraota	Conventional
Brown (small)	*lpa*-1	*lpa*
Brown (small)	*lpa*-2	*lpa*

**Table 2:** Overview of iron, zinc and phytic acid content for the seven dry bean varieties that were selected for this study. The varieties were grouped as either conventional, biofortified or *lpa* varieties.

Cultivars	Group	Iron			Zinc			Total Phytic Acid
		(μg·g^−1^)			(μg·g^−1^)			(mg·g^−1^)
Calima	Conventional	54.39	±	0.10 d	30.17	±	0.46 cd	17.28 ± 1.65 b
Caraota	Conventional	60.48	±	1.41 c	31.93	±	0.16 c	14.83 ± 0.11 c
**Average conventional**		**57.44**	**±**	**3.61** **C**	**31.05**	**±**	**1.06 B**	**16.05 ± 1.71 A**
BIO 101	Biofortified	90.23	±	0.59 a	43.52	±	0.37 a	14.83 ± 0.30 c
BIO 107	Biofortified	87.27	±	0.57 a	36.79	±	0.23 b	21.00 ± 0.28 a
ICTA Chorti	Biofortified	87.87	±	2.55 a	37.00	±	0.14 b	20.05 ± 0.35 a
**Average biofortified**		**88.46**	**±**	**1.84 A**	**39.10**	**±**	**3.43 A**	**18.62 ± 2.98 A**
*lpa-1*	*lpa*	73.50	±	1.16 b	28.72	±	0.38 d	1.05 ± 0.01 d
*lpa* -2	*lpa*	75.44	±	1.78 b	31.63	±	1.20 c	1.10 ± 0.04 d
**Average *lpa***		**74.47**	**±**	**1.66 B**	**30.17**	**±**	**1.83 B**	**1.07 ± 0.04 B**

The results of ANOVA analysis using PosdHoc Tukey HSD test were validated using General Linear (GL) analysis followed by multiple comparison of means by Tukey contract (Posthoc). The *p*-values were adjusted to a single-step method. SD = standard deviation for each of the genotypes and groups. The letters in bold capitals (A,B,C) indicate differences on group level. The letters in lowercase (a,b,c,d) indicate significant differences between varieties.

**Table 3:** Cooking times of the seven different bean varieties using the Mattson Cooker, soaked for 16 h at room temperature.

Cultivars	Cooking Time ± SD (min)		
Calima	52.1	±	0.8 bc
Caraota	51.8	±	3.2 abc
BIO 101	33.8	±	1.2 d
BIO 107	41.9	±	1.5 bcd
ICTA Chorti	62.7	±	3.1 ab
*lpa-1*	55.4	±	10.8 abc
*lpa-2*	58.8	±	14.4 ab

The ANOVA analysis with TukeyHSD test was validated by linear mixed model (LMM) analysis. Analysis of variance indicated that bean variety had the most significant effect for the model fit. The lower case letters (a,b,c) indicate significant differences in cooking times between the different varieties assessed in triplicate, as analysed by pairwise multiple comparison of means by Tukey contract (Post hoc) at confidence level 0.95.

**Table 4:** True and apparent retention (% ± SD) for iron of three groups of beans after five different processing steps.

Processing Step	Group	True Retention (% ± SD)		Apparent Retention (% ± SD)	
		Soaked	Non-Soaked	Soaked	Non-Soaked
*Soaking*	Conventional	108.4 ± 7.0 a	NA	NA	NA
	Biofortified	97.8 ± 2.7 b	NA	NA	NA
	*lpa*	98.8 ± 5.6 b	NA	NA	NA
*Boiling*	Conventional	87.8 ± 2.0 a	91.3 ± 9.8 a	119.3 ± 3.2 a	119.6 ± 11.3 a
	Biofortified	86.6 ± 2.5 a	82.1 ± 3.5 ab	118.2 ± 3.6 a	112.4 ± 3.14 a
	*lpa*	77.2 ± 3.4 b	77.7 ± 5.3 b	105.7 ± 3.8 b	104.8 ± 6.0 b
*Refrying*	Conventional	97.9 ± 11.5 a	104.5 ± 5.3 a	100.5 ± 9.7 a	97.7 ± 5.3 a
	Biofortified	93.4 ± 5.1 a	91.2 ± 2.9 b	97.0 ± 2.8 a	93.0 ± 2.7 a
	*lpa*	87.3 ± 6.7 a	92.3 ± 3.7 b	91.4 ± 6.4 a	93.6 ± 3.1 a

The ANOVA analysis with TukeyHSD test was validated by linear mixed model (LMM) analysis. The lower case letters (a,b,c) indicate significant differences between the different groups in the column (per treatment). (*p* < 0.05). Values are average ± standard deviation. NA = Not Applicable.

**Table 5:** True and apparent retention (% ± SD) for zinc of three groups of beans after five different processing steps.

Processing Step	Group	True Retention (% ± SD)		Apparent Retention (% ± SD)	
		Soaked	Non-Soaked	Soaked	Non-Soaked
*Soaking*	Conventional	99.4 ± 2.3 a	NA	NA	NA
	Biofortified	93.3 ± 4.7 a	NA	NA	NA
	*lpa*	93.6 ± 3.2 a	NA	NA	NA
*Boiling*	Conventional	75.3 ± 4.3 a	84.0 ± 5.6 a	89.0 ± 5.4 a	95.8 ± 5.7 a
	Biofortified	77.9 ± 2.8 a	81.1 ± 6.3 a	92.3 ± 2.8 a	96.2 ± 4.4 a
	*lpa*	41.2 ± 4.2 b	46.4 ± 3.0 b	49.3 ± 4.5 b	54.6 ± 2.7 b
*Refrying*	Conventional	86.6 ± 7.6 a	100.0 ± 4.0 a	77.4 ± 5.2 a	81.4 ± 4.0 a
	Biofortified	85.6 ± 2.5 a	91.3 ± 3.7 b	77.3 ± 3.3 a	80.8 ± 3.9 a
	*lpa*	63.5 ± 6.6 b	77.7 ± 3.1 c	58.1 ± 5.6 b	68.8 ± 2.6 b

The ANOVA analysis with TukeyHSD test was validated by linear mixed model (LMM) analysis. The lower case letters (a,b,c) indicate significant differences between the different groups in the column (per treatment). (*p* < 0.05). ANOVA analysis of zinc true retention, and apparent retention variants was performed using transformed data (quadratic transformation based on power lambda λ). Values are averages ± standard deviation. NA = Not Applicable.

**Table 6:** True and apparent retention (% ± SD) for phytic acid of three groups of beans after five different processing steps.

Processing Step	Group	True Retention (% ± SD)		Apparent Retention (% ± SD)	
		Soaked	Non-soaked	Soaked	Non-soaked
*Soaking*	Conventional	65.7 ± 16.7 b	NA	NA	NA
	Biofortified	88.5 ± 9.1 a	NA	NA	NA
	*lpa*	83.9 ± 4.7 a	NA	NA	NA
*Boiling*	Conventional	49.9 ± 2.1 c	64.0 ± 9.7 b	59.0 ± 3.2 c	73.2 ± 12.0 b
	Biofortified	58.6 ± 5.0 b	62.8 ± 2.9 b	69.5 ± 6.2 b	74.7 ± 5.3 b
	*lpa*	72.1 ± 5.8 a	85.9 ± 4.7 a	86.3 ± 6.1 a	101.3 ± 6.5 a
*Refrying*	Conventional	59.7 ± 9.5 a	77.3 ± 11.7 ab	53.6 ± 10.1 b	62.9 ± 10.1 ab
	Biofortified	65.6 ± 7.2 a	72.7 ± 5.6 b	59.0 ± 4.1 ab	64.3 ± 4.0 b
	*lpa*	73.5 ± 5.4 a	86.9 ± 3.9 a	67.2 ± 4.9 a	77.0 ± 4.0 a

The ANOVA analysis with TukeyHSD test was validated by linear mixed model (LMM) analysis. The lower case letters (a,b,c) indicate significant differences between the different groups in the column (per treatment). (*p* < 0.05). Values are averages ± standard deviation. NA = Not Applicable.

**Table 7:** Contribution of beans to iron and zinc EAR for children and adults for three groups (conventional, biofortified and *lpa*) of beans and two preparation methods (boiled and refried).

Mineral	Preparation Method	Population	Conventional	Biofortified	*lpa*
			beans	beans	beans
Iron	Boiled	Children 4–6	29%	44%	34%
		Adult women	27%	40%	31%
	Refried	Children 4–6	31%	46%	38%
		Adult women	29%	43%	35%
Zinc	Boiled	Children 4–6	15%	19%	8%
		Adult women	15%	19%	8%
	Refried	Children 4–6	16%	21%	13%
		Adult women	17%	21%	13%

Based on fresh weight multiplied by portion size. One portion is defined as 55 g and 100 g of cooked and 68.75 g and 125 g of refried beans for respectively children and adult women.

**Table 8:** Phytic acid to iron and zinc molar ratios for three groups of beans (conventional, biofortified and *lpa*) and two preparation methods (boiled and refried).

Preparation Method	Group	Phytic Acid to Iron Molar Ratio	Phytic Acid to Zinc Molar Ratio
Boiled	Conventional	15	36
	Biofortified	13	36
	*lpa*	1	6
Refried	Conventional	16	37
	Biofortified	13	35
	*lpa*	1	4

## Supplementary Materials

The following are available online at https://www.mdpi.com/2072-6643/12/3/658/s1, Figure S1: The low phytic acid bean lines used in this study (*lpa-1* and *lpa-2*) were generated at the International Centre of Tropical Agriculture (CIAT), Colombia, from the line *lpa-127-4* which is a BC2 (backcross 2) progeny arising from the backcrossing of the original *lpa* mutant *lpa280-10* (a homozygous monogenic recessive *lpa* mutant line originally obtained by EMS mutagenesis, [30]) with the bean cultivar BAT93.

## Appendix A

**Table A1:** Fresh and dry weight (μg·g^−1^ ± SD) for iron of three groups of beans after five different processing steps.

Processing Step	Group	Fresh Weight (μg·g^−1^ ± SD)		Dry Weight (μg·g^−1^ ± SD)	
		Soaked	Non-Soaked	Soaked	Non-Soaked
*Soaking*	Conventional	27.50 ± 1.42 b	NA	64.18 ± 2.66 a	NA
	Biofortified	39.05 ± 1.97 a	NA	91.28 ± 3.11 a	NA
	*lpa*	30.48 ± 1.52 b	NA	78.48 ± 4.57 c	NA
*Boiling*	Conventional	22.48 ± 1.92 c	21.38 ± 2.12 b	59.70 ± 5.52 c	59.60 ± 5.08 c
	Biofortified	32.05 ± 1.14 a	32.98 ± 1.43 a	90.75 ± 2.18 a	86.32 ± 1.65 a
	*lpa*	26.08 ± 1.69 b	24.65 ± 0.96 b	68.75 ± 3.22 b	68.15 ± 4.59 b
*Refrying*	Conventional	19.50 ± 2.63 c	17.95 ± 1.96 b	50.20 ± 5.34 b	48.90 ± 5.38 c
	Biofortified	27.78 ± 1.09 a	27.45 ± 0.39 a	74.48 ± 1.66 a	71.42 ± 1.78 a
	*lpa*	23.88 ± 0.53 b	21.50 ± 1.70 c	59.43 ± 4.83 b	60.88 ± 2.68 b

The ANOVA analysis with TukeyHSD test was validated by linear mixed model (LMM) analysis. The lower case letters (a,b,c) indicate significant differences between the different groups in the column (per treatment) (*p* < 0.05). Values are average ± standard deviation. NA = Not Applicable.

**Table A2:** Fresh and dry weight (μg·g^−1^ ± SD) for zinc of three groups of beans after five different processing steps.

Processing Step	Group	Fresh Weight (μg·g^−1^ ± SD)		Dry Weight (μg·g^−1^ ± SD)	
		Soaked	Non-Soaked	Soaked	Non-Soaked
*Soaking*	Conventional	13.65 ± 0.55 b	NA	31.85 ± 0.68 a	NA
	Biofortified	16.45 ± 1.88 a	NA	38.47 ± 4.03 b	NA
	*lpa*	11.7 ± 0.81 b	NA	30.13 ± 2.29 a	NA
*Boiling*	Conventional	9.90 ± 0.93 b	11.20 ± 0.8 b	27.70 ± 2.50 b	29.73 ± 2.09 b
	Biofortified	13.12 ± 1.00 a	14.00 ± 1.46 a	36.07 ± 2.77 a	37.53 ± 2.84 a
	*lpa*	5.38 ± 0.69 c	6.33 ± 0.67 c	14.88 ± 2.08 c	16.53 ± 1.70 c
*Refrying*	Conventional	8.60 ± 0.68 b	10.10 ± 0.96 b	24.03 ± 1.83 b	25.28 ± 1.87 b
	Biofortified	11.12 ± 1.12 a	12.27 ± 0.83 a	30.23 ± 2.46 a	31.52 ± 1.71 a
	*lpa*	6.35 ± 0.94 c	8.15 ± 0.45 c	17.60 ± 2.64 c	20.80 ± 1.58 c

The ANOVA analysis with TukeyHSD test was validated by linear mixed model (LMM) analysis. The lower case letters (a,b,c) indicate significant differences between the different groups in the column (per treatment) (*p* < 0.05). Values are averages ± standard deviation. NA = Not Applicable.

**Table A3:** Fresh and dry weight (mg·g^−1^ ± SD) for total phytic acid of three groups of beans after five different processing steps.

Processing Step	Group	Fresh Weight (mg·g^−1^ ± SD)		Dry Weight (mg·g^−1^ ± SD)	
		Soaked	Non-Soaked	Soaked	Non-Soaked
*Soaking*	Conventional	4.60 ± 0.91 b	NA	10.74 ± 1.91 b	NA
	Biofortified	6.98 ± 0.83 a	NA	16.04 ± 1.9 a	NA
	*lpa*	0.40 ± 0.00 c	NA	0.96 ± 0.03 c	NA
*Boiling*	Conventional	3.38 ± 0.21 b	4.38 ± 0.42 a	9.47 ± 0.61 b	11.66 ± 1.06 a
	Biofortified	4.70 ± 0.70 a	5.03 ± 1.08 a	12.81 ± 1.7 a	13.81 ± 3.48 a
	*lpa*	0.33 ± 0.05 c	0.40 ± 0.00 b	0.92 ± 0.09 c	1.08 ± 0.04 b
*Refrying*	Conventional	3.03 ± 0.33 a	3.98 ± 0.43 a	8.53 ± 0.91 a	10.02 ± 0.82 a
	Biofortified	4.03 ± 0.75 a	4.3 ± 0.84 a	10.79 ± 2.16 a	11.24 ± 2.35 a
	*lpa*	0.28 ± 0.05 b	0.3 ± 0.00 b	0.72 ± 0.07 b	0.82 ± 0.04 b

The ANOVA analysis with TukeyHSD test was validated by linear mixed model (LMM) analysis. The lower case letters (a,b,c) indicate significant differences between the different groups in the column (per treatment). (*p* < 0.05). Values are averages ± standard deviation. NA = Not Applicable.
