# Supplementary material for: Iron, Zinc and Phytic Acid Retention of Biofortified, Low Phytic Acid, and Conventional Bean Varieties When Preparing Common Household Recipes
Source: Nutrients. 2020 Feb 28;12(3):658. doi: 10.3390/nu12030658 (PMC7146319; doi:10.3390/nu12030658)
Supplement: Supplementary file 1 [file nutrients-12-00658-s001.pdf]

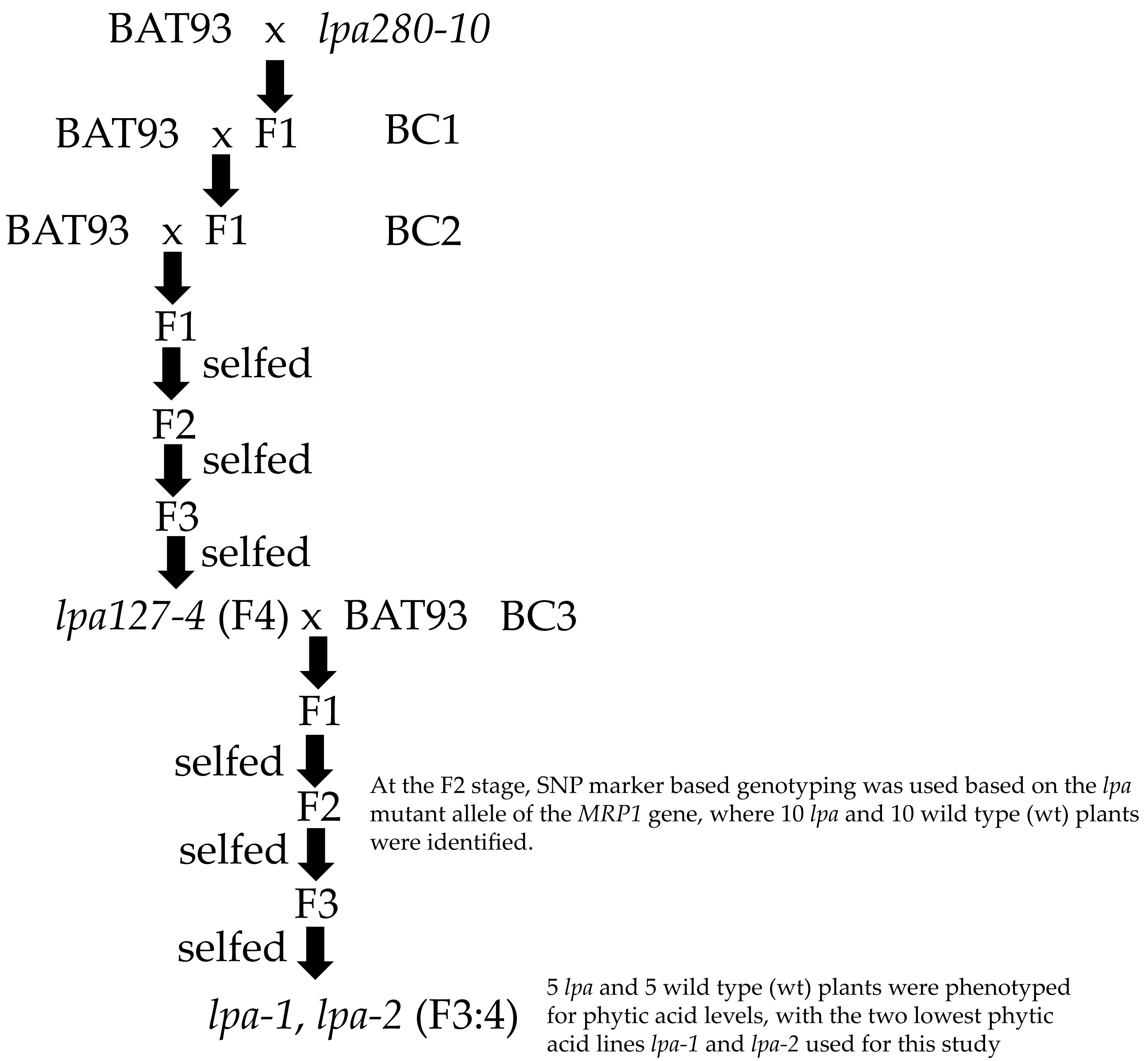

Supplementary Figure 1. The low phytic acid bean lines used in this study (*lpa-1* and *lpa-2*) were generated at the International Centre of Tropical Agriculture (CIAT), Colombia, from the line *lpa-127-4* which is a BC2 (backcross 2) progeny arising from the backcrossing of the original *lpa* mutant *lpa280-10* (a homozygous monogenic recessive *lpa* mutant line originally obtained by EMS mutagenesis, [30]) with the bean cultivar BAT93. The line *lpa-127-4* was further backcrossed to BAT93 and the two low phytic acid *lpa* lines, *lpa-1* and *lpa-2*, were identified as having the lowest phytic acid content out of the lines screened across the F4 generation population.
